# A Scoping Review of Social Needs and Networks of Older Women Living With HIV

**DOI:** 10.1093/geroni/igaf122.942

**Published:** 2025-12-31

**Authors:** Jasmine Manalel, Alvin Gao

**Affiliations:** Hunter College, CUNY, New York, North Carolina, United States; Hunter College, New York, New York, United States

## Abstract

Older women living with HIV require adequate support from their social networks to meet their informal care needs and promote psychosocial well-being as they age. Understanding the social resources available to them and their effectiveness in meeting care needs is essential. The purpose of this scoping review is to summarize findings and identify gaps in research on informal care needs and networks of older women living with HIV. We conducted a scoping review using key search terms related to aging, women, HIV, social relationships, and care needs. We included English-language empirical work that focused on women with HIV aged 45 and older and explicitly discussed social relationships or informal care needs. Thematic content analysis was used to analyze the data. Out of 3,108 screened articles, 25 were included. Eighteen studies were based in the United States. Selected articles included 17 qualitative studies (including two longitudinal), 6 cross-sectional quantitative studies, and 3 mixed-methods studies. Guided by the convoy model of social relations, we focused on network structure, support, and isolation. The most common relationships discussed were with romantic partners (13 studies) and children (9 studies). Social relations and informal care needs were shaped by caregiving roles (13 studies), HIV stigma (13 studies), and had wide-ranging impacts on psychosocial outcomes (4 studies). Findings highlighted that social relationships can be both a source of stress and resilience. This review documented the complexity of the care networks for women aging with HIV, shaped by stigma and changing social roles, and their effects on well-being.

